# Precision-engineering of subunit vaccine particles for prevention of infectious diseases

**DOI:** 10.3389/fimmu.2023.1131057

**Published:** 2023-02-03

**Authors:** Shuxiong Chen, Saranya Pounraj, Nivethika Sivakumaran, Anjali Kakkanat, Gayathri Sam, Md. Tanvir Kabir, Bernd H. A. Rehm

**Affiliations:** ^1^ Centre for Cell Factories and Biopolymers (CCFB), Griffith Institute for Drug Discovery, Griffith University, Nathan, QLD, Australia; ^2^ Menzies Health Institute Queensland (MHIQ), Griffith University, Gold Coast, QLD, Australia

**Keywords:** particulate vaccine, cross-presentation, post-translational modification, dendritic cells, protective immunity, infectious diseases

## Abstract

Vaccines remain the best approach for the prevention of infectious diseases. Protein subunit vaccines are safe compared to live-attenuated whole cell vaccines but often show reduced immunogenicity. Subunit vaccines in particulate format show improved vaccine efficacy by inducing strong immune responses leading to protective immunity against the respective pathogens. Antigens with proper conformation and function are often required to induce functional immune responses. Production of such antigens requiring post-translational modifications and/or composed of multiple complex domains in bacterial hosts remains challenging. Here, we discuss strategies to overcome these limitations toward the development of particulate vaccines eliciting desired humoral and cellular immune responses. We also describe innovative concepts of assembling particulate vaccine candidates with complex antigens bearing multiple post-translational modifications. The approaches include non-covalent attachments (e.g. biotin-avidin affinity) and covalent attachments (e.g. SpyCatcher-SpyTag) to attach post-translationally modified antigens to particles.

## Immunologic properties of soluble and particulate vaccines

Subunit vaccines contain selected immunogenic components of the pathogen to elicit an immune response (1, 2). Particulate vaccines involve the attachment of the antigens to microcarriers through chemical adsorption, encapsulation, conjugation, or biological self-assembly for enhanced delivery and induction of an efficient immune response (3, 4). Soluble vaccines are weakly immunogenic when compared to their insoluble counterparts such as when displayed on particulate carrier (5). Due to low immunogenicity, soluble vaccines often require the administration of multiple boosts (6, 7). The use of immunostimulatory adjuvants along with soluble vaccines would improve the immune responses but potentially increases the overall vaccine production cost (8). However, antigens immobilized on particulate carrier exhibit enhanced immunogenicity (9–11). Some particulate carriers themselves act as adjuvants resulting in enhanced and targeted immune responses. Particulate carrier also allow the codelivery of adjuvants and multiple antigens to the same antigen presenting cells (APCs) (12, 13). Therefore, particulate vaccines are promising and potent antigen delivery systems to overcome the low immunogenicity of soluble subunit vaccine formulations.

**Figure 1:**
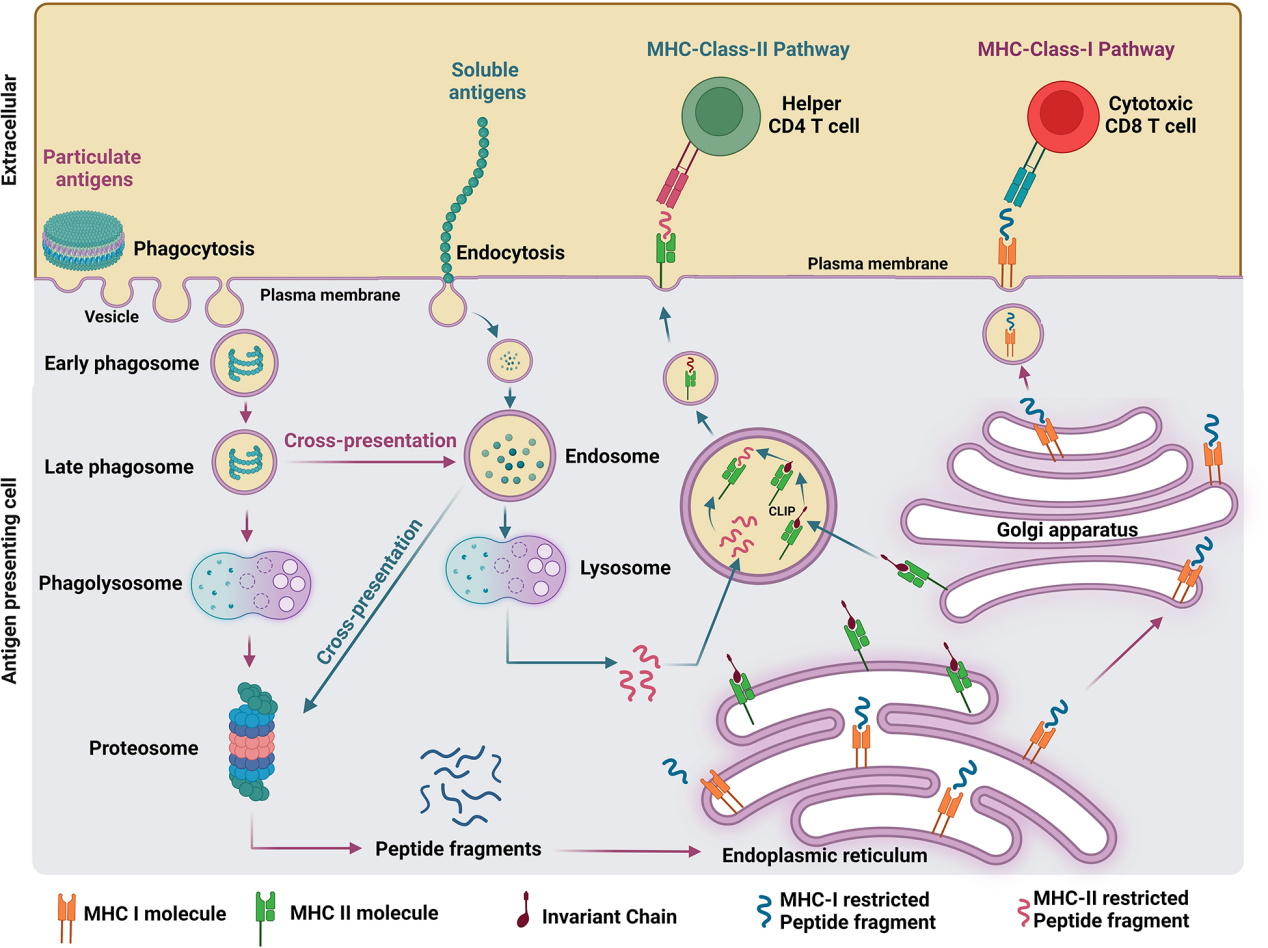
Comparison of antigen processing pathways elicited by soluble and particulate antigen formulations.

The proposed mechanism of antigen processing elicited by soluble and particulate vaccine formulations is illustrated in **Figure 1**. Soluble antigens can be internalized *via* endocytosis (14) and exclusively presented by major histocompatibility complex (MHC) II machinery in the endosomes, whereas particulate antigens above 500 nm can be phagocytosed by APCs into the phagosomes and presented by both MHC class I and II machineries in the cytosol (7). The maturation of the phagosome occurs after the formation of nascent phagosomes (pH 7.4) containing the engulfed particulate antigens which are then sequentially trafficked into progressive acidified compartments called early phagosome (pH 6), late phagosome (pH 5.5), and phagolysosomes (pH 5) (15). The efficient degradation of antigens into smaller peptides occurs in the phagolysosomes which contain a variety of digestive enzymes such as proteases, lipases, and glycosidases without degrading the epitopes (16). Further degradation of antigens into smaller polypeptides occurs in the cytosol by a protein complex called proteosomes (17). The degraded protein fragments will be transported into the endoplasmic reticulum, where the folding and assembly of the heavy and light chain of MHC molecules occurs and facilitates the MHC and peptide binding (18). Finally, the peptide-loaded MHC complex gets transported to the cell surface through the Golgi apparatus and attracts both CD4^+^ T cells and CD8^+^ T cells with specific receptors to mount a cell-mediated immune response eliminating the damaged/infected cells displaying corresponding peptide fragments (19). MHC class I and II pathways are usually involved presenting peptides from intracellular or extracellular pathogens, respectively. However, dendritic cells (DCs) possess the ability to divert peptides derived from extracellular pathogens to cytotoxic CD8^+^ T cells *via* the MHC class I presentation pathway (20). A phagosome is a key organelle in antigen cross-presentation which primarily induces cytotoxic CD8^+^ T cell responses. However, such responses mediated by the phagosome route are not achievable by endocytosed soluble antigens (21). In addition, B cell receptors (BCRs) are uniformly distributed on B cell surface in the absence of pathogen invasion. However, BCRs are brought together to bind multiple copies of antigens on the invading pathogen’s surface. This process is also called BCR cross-linking, required for B cell activation. Repetitive display of antigens on particles facilitates efficient recognition and BCR cross-linking, which allow strong B cell activation and antigen uptake for presentation to CD4^+^ T cells. It results in inducing higher levels of neutralizing antibodies and functional cellular immune responses than achieved by soluble antigens present in most subunit vaccines (22).

Soluble antigens are engulfed by APCs through endocytosis and get presented to endosomes. Consequently, endosomes fuse with lysosomes and degrade the antigens into peptide fragments restricted to both MHC class I and II. MHC class II specific peptides bind to MHC class II molecules by replacing the class II-associated invariant chain peptide (23). This peptide-MHC class II complex will get presented to the cell surface and activates naïve CD4^+^ T cells. However, if the peptides are MHC class I specific, they will get cross-presented to proteosomes and follows MHC class-I pathway to expand cytotoxic CD8^+^ T cells (14). Even though the cross-presentation of soluble antigen was detected *in vivo*, it is not very efficient in generating strong immune responses (24). However, unlike soluble antigens, particulate antigens are sustainably presented by APCs in large quantities for a prolonged time resulting in enhanced immunogenicity (25).

## Antigen delivery platforms and their immunological properties

### Virus-like particles (VLPs)

**Figure 2:**
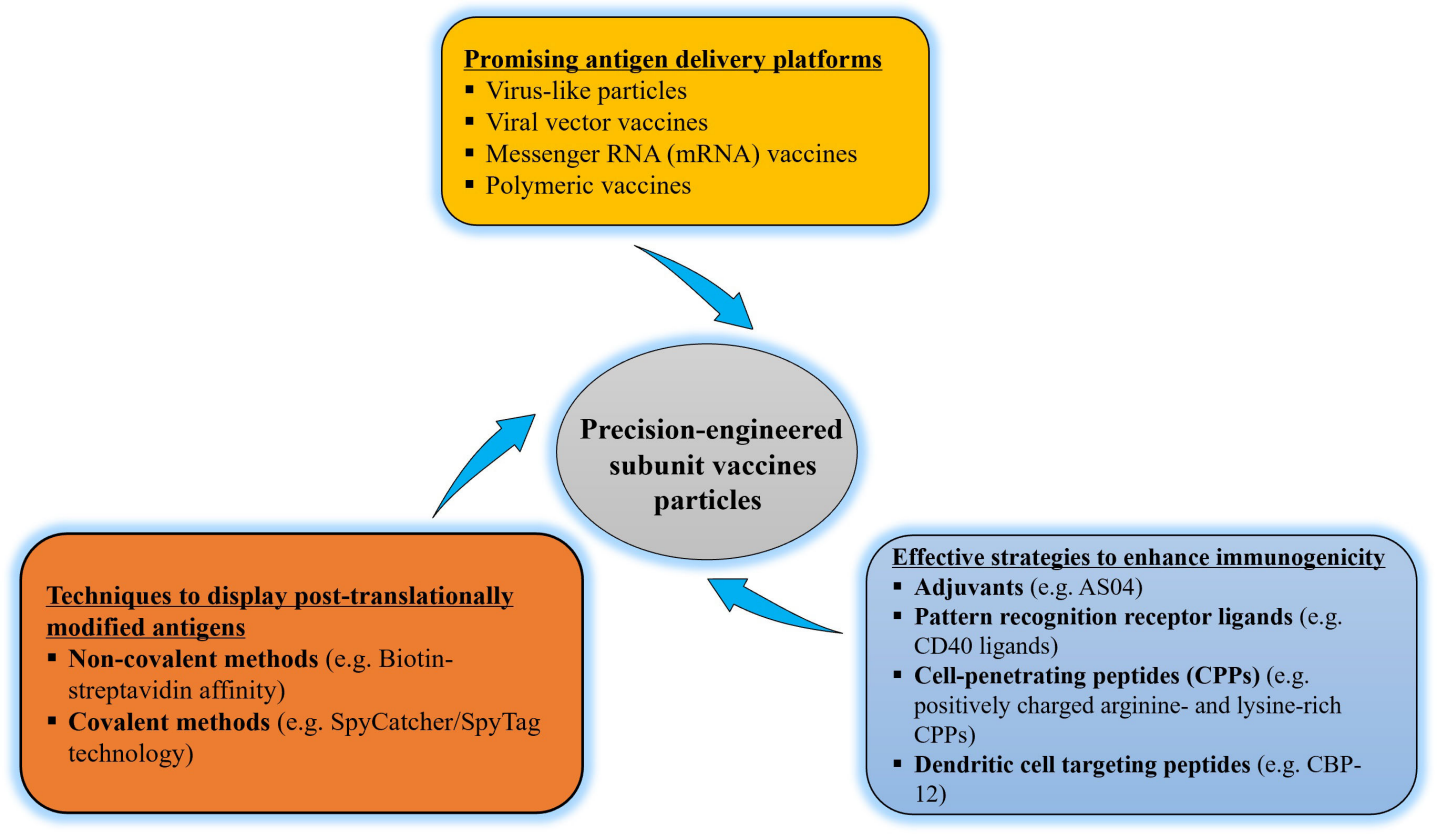
Flow diagram of precision-engineering of subunit vaccine particles.

Virus-like particles (VLPs) are nanostructures made up of self-assembled virus proteins. VLPs do not contain viral genomes and thus they do not have the capacity of infecting the host cell (26). VLPs can be generated by using bacterial, yeast, insect, plant, and mammalian cells (27). It has been reported that VLPs can serve as carriers for the delivery of various biomaterials and nanomaterials including vaccines (**Figure 2**) (26, 28, 29). Currently, VLP-based vaccines against human papillomavirus, hepatitis B virus and malaria are commercially available (**Table 1**) (26, 30).

**Table 1:** Antigen delivery platforms and their immunological properties.

	Virus-like particles	Viral vector vaccines	Messenger RNA (mRNA) vaccines	Polymeric vaccines
**Components**	Nanostructures made up of self-assembled virus proteins but do not contain viral genomes	Comprise a harmless, modified, and unrelated virus that delivers foreign genetic material (DNA)	Deliver a transcript of interest that encodes a target antigen	Natural and synthetic polymers are used to encapsulate a range of vaccine components
**Advantages**	Safe, presentation of multiple epitopes, highly immunogenic, induce both cellular and humoral immune responses	Sustained immune responses, enhanced immunogenicity without the need for adjuvant co-administration, powerful cellular and antibody responses	Rapid development, and a cell-free manufacturing process, safer and more effective than DNA-based vaccines, do not require nuclear entry, do not pose a risk of integration into the host genome, intrinsic self-adjuvant properties, exert potent cellular and humoral immune responses	Induction of enhanced levels of antigen-specific antibodies, extended antigen circulation, co-loading of antigens, elevated level of cytokine release, strong cellular and humoral immune responses owing to their unique properties including their pathogen mimicking size, controllable lipophilicity/hydrophobicity, modifiable surfaces, and high surface-to-volume ratios, capable of delivering a wide range of antigenic molecules
**Disadvantages**	Instability, high manufacturing cost	Expensive, highly complex, pose a risk for the environment and human health	Limited transfection efficiency, degradation of free mRNA *via* nucleases, degradation of exogenous naked mRNA in the endolysosomal compartments	Some chemically synthesized polymer materials (such as polyurethane) can’t be naturally degraded and may also cause environmental pollution, some inorganic material carriers show low immunogenicity and poor biodegradability
**Examples of licensed vaccines**	Human papillomavirus, hepatitis B virus, and malaria vaccines	Johnson & Johnson’s Janssen (J&J/Janssen) COVID-19 vaccine, Ebola virus vaccine (Ervebo)	Moderna (mRNA-1273) and Pfizer-BioNTech (BNT162b2) COVID-19 vaccines	Further clinical studies are needed for regulatory approval
**References**	(26, 28, 30–32)	(33–35)	(36–46)	(47–51)

VLP-based vaccines are highly immunogenic and can induce both cellular and humoral immune responses and the use of adjuvants enhances their immunogenicity (32). VLPs were found to strongly activate DCs (32). DCs are well-known as the most effective APCs and have the ability to activate both naϊve and memory immune responses (52). Activation of DCs takes place owing to the binding of VLPs with the receptors that are present on the surface of DCs known as pattern recognition receptors (PRRs) such as Toll-like receptors (TLRs) (32, 53). VLP-based vaccines are then recognized and internalized by DCs and get presented to CD4^+^ helper T cells and cytotoxic CD8^+^ T cells *via* MHC class II and MHC class I molecules, respectively (32, 54). VLPs allow presentation of multiple copies of epitopes on their surface, which mediate cross-linking of B cell receptors and subsequent priming B cells toward generation of antibodies even without the help of CD4^+^ T helper cells (32). VLP-based vaccines are regarded safer as compared to attenuated or inactivated viruses. Nonetheless, instability of VLPs can compromise their immunogenicity and the production cost of some VLP-based vaccines can be high because of their low yields (28, 31).

### Viral vector vaccines (VVVs)

Viral vectors are considered as an advanced approach for the development of vaccines. Viral vectors have been used to produce vaccines against multiple infectious diseases including SARS-CoV-2, Ebola, Malaria, and HIV (34, 55). Viral vector vaccines (VVVs) comprise a harmless, modified, and unrelated virus that delivers foreign genetic material (DNA) to human cells, which then produce the pathogen-specific antigens encoded by the DNA (33). Moreover, VVVs offer multiple advantages as compared to other vaccine platforms including long-lasting immune responses, high immunogenicity without the need for adjuvant co-administration, and strong cellular and antibody responses (34).

Viral vectors such as based on adenovirus, poxvirus, influenza viruses, and measles virus are currently widely considered for the development of VVVs (56). Among them, the adenoviral vector has been used extensively to develop COVID-19 vaccines (56, 57). VVVs can induce long-lasting and strong cellular responses to eliminate virus-infected cells. Cytotoxic CD8^+^ T cells rapidly proliferate and differentiate in response to antigens, while cell-mediated immunity induces natural killer cells and macrophages to eliminate intracellular pathogens (58). On the other hand, most of the VVVs can induce powerful humoral immunity, however it has been observed that immunogen design can affect VVV-mediated humoral immunogenicity (58). For example, Huang et al. (59) revealed that immunizing mice or hamsters with SARS-CoV-2 spike proteins lacking glycan shields induced potent and broadly reactive immune responses. Despite many advantages, the production process of VVVs is comparatively expensive and highly complex. Moreover, VVVs could pose a risk for the environment and human health (35).

### Messenger RNA (mRNA) vaccines

Messenger RNA (mRNA) vaccines have already been found highly effective against various infectious diseases (36, 60). There are several advantages offered by mRNA vaccines including rapid development, and a cell-free manufacturing process (36). mRNA vaccine technology involves delivering a transcript of interest that encodes a target antigen (37). There are two categories of mRNA vaccines including self-amplifying and non-replicating mRNA vaccines (61). mRNA vaccines precisely encode the specific antigen of interest, and then cells that take up the mRNA can translate it into proteins (62, 63). Subsequently, the immune system mounts robust adaptive immune responses against the target proteins (63). mRNA vaccines are safer and more effective than DNA-based vaccines, since mRNA vaccines do not require nuclear entry and they do not pose a risk of integration into the host genome (38–40). In addition, since the outbreak of the COVID-19 pandemic, there is a growing research interest in mRNA vaccines because of their capacity to trigger strong immune responses, rapid development, and simple manufacturing process (64). Currently approved mRNA COVID-19 vaccines include Moderna (mRNA-1273) and Pfizer-BioNTech (BNT162b2) (41–43).

mRNA vaccines contain intrinsic self-adjuvant properties and exert potent cellular and humoral immune responses (65). Hence mRNA vaccines have the potential to induce both innate and adaptive immune responses. mRNA vaccines also generated strong immune responses in experimental animal models of various infectious diseases including rabies, Zika virus disease, and influenza (66, 67). Following the delivery of mRNA vaccines in the body, they are taken up by APCs such as DCs or macrophages. Subsequently, they escape the endosome and enter into the cytosol, where mRNA is translated into protein by the ribosome (68). In the lymph node, antigen derived peptides are presented *via* MHC class I or II to cytotoxic CD8^+^ T cells or CD4^+^ T cells, which ultimately results in the activation of cell-mediated and humoral immune responses (69). However, there are some drawbacks of mRNA vaccines that need to be addressed to develop more effective, stable, and safe mRNA vaccines. These drawbacks include limited transfection efficiency, degradation of free mRNA *via* nucleases, and degradation of exogenous naked mRNA in the endolysosomal compartments (44–46). In addition, rare evidence was found that severe anaphylaxis and myocarditis occurred after immunization with mRNA-containing lipid nanoparticles (mRNA-LNP). It is believed that the lipid component, polyethylene glycol (PEG) 2000, in the vaccine formulation causes anaphylactic shock, especially in those who already have pre-existing anti-PEG antibodies (70).

### Polymeric vaccines

There is a growing interest in polymer-based vaccines because of their several beneficial properties including induction of enhanced levels of antigen-specific antibodies, extended antigen circulation, co-loading of antigens, elevated level of cytokine release, and potent cellular immune responses (47). Polymer-based particles can be used as vaccine platforms to deliver numerous antigenic molecules including nucleic acids, carbohydrates, cell lysates, lipopeptides, peptides, and proteins (71). Polymer-based particles have great potential in the prevention and treatment of infectious diseases (51). Lipid- and polymer-based NPs have already been extensively studied in vaccine development. NPs have the capacity to induce strong cellular and humoral immune responses owing to their unique properties including their pathogen mimicking size, controllable lipophilicity/hydrophobicity, modifiable surfaces, and high surface-to-volume ratios (48).

There are two major groups of biodegradable polymers such as natural and synthetic polymers. A range of both synthetic and natural biodegradable polymers have already been studied for the development of vaccines. Naturally occurring biomaterials including various proteins including silk, fibrin, and collagen, and several polysaccharides including hyaluronic acid derivatives, chitosan, alginate, and starch have widely been studied to prepare particulate vaccine formulations (72). On the other hand, many synthetic biodegradable polymers including polyanhydrides, polyphosphazene, and various polyesters including poly(lactic-co-glycolic acid), polyglycolide, polycaprolactone, and polylactides have been studied as potential vaccine delivery systems (73).

Polyhydroxyalkanoates (PHAs) have been identified as biocompatible and biodegradable biopolymers and that are produced by a range of bacteria serving as energy and carbon storage materials (74). PHAs have drawn a lot of attention because of their potential as particulate vaccines in delivering various antigens from diverse pathogens (75). Furthermore, PHA particles displaying one or more antigens already exhibited improved cell-mediated and humoral immune responses when compared to the corresponding soluble subunit vaccines (49). Extensive animal trials have already confirmed that PHA particle vaccine candidates are safe and have the potential to induce robust and protective immune responses against bacterial and viral pathogens (**Table 2**) (11, 76, 78, 79, 85). Despite the great potential of polymeric nanostructured vaccines, further clinical studies are needed towards regulatory approval (51).

**Table 2:** PHA particle vaccines with protective immunity against various infectious pathogens.

Pathogen	Selected antigen/epitopes	Animal model	Humoral responses	T cell responses	Protective Immunity	Reference
*Streptococcus suis*	SSU1915, SSU1355, SSU0185, SSU1215, SSU1773	Mouse	IgG, IgM		Yes	(76)
*Mycobacterium tuberculosis*	H4, H28	Mouse	IgG, IgG1, IgG2c	Th1, Th2, Th17	Yes	(11)
*Plasmodium falciparum*	B/T cell epitopes of CSP	Sheep	IgG		Yes	(77)
*SARS-CoV-2*	S1, N, SM epitopes	Mouse	IgG, IgG1, IgG2c	Th1, Th2	Yes	(78)
		Hamster	IgG neutralizing ab			
*Pseudomonas aeruginosa*	Oprl, OprF, AlgE, OprL, PopB, PilA, PilO, FliC, Hcp1, CdrA	Mouse	IgG, IgG1, IgG2a, IgG2c Opsonophagocytic antibodies	Th1, Th2	Yes	(79, 80)
*Hepatitis C virus*	HCc	Mouse	IgG1, IgG2c	Th1, Th2, Th17	Yes	(81)
*Mycobacterium tuberculosis*	Ag85A and ESAT-6	Mouse		Th1, Th2, Th17	Yes	(82)
*Streptococcus pneumoniae*	PsaA, Ply, Serotype 19F CPS	Mouse	IgG1, IgG2c Opsonophagocytic antibodies	Th1, Th2	Yes	(83)
*Neisseria meningitidis*	Serogroup C CPS, NadA, fHbp	Mouse	IgG1, IgG2a, IgG2b, IgG3	Th1, Th2, Th17	Yes	(84)

In a recent study, the immunogenic carrier protein, CRM197 (non-toxic variant diphtheria toxin) was engineered for the assembly of antigen-containing submicron-sized particles (86). Chen et al. (2022) developed a bioprocess for high-yield production *via* efficient assembly inside engineered endotoxin-free *Escherichia coli.* These particles were ambient-temperature stable and were precision engineered to serve as vaccine candidates that induce protective immunity against SARS-CoV-2, *Mycobacterium tuberculosis* and group A Streptococcus, respectively. The study demonstrated the utility and versatility of engineering immunogenic carrier proteins, that have been approved in vaccine formulation, into a synthetic particulate vaccine for induction of functional humoral and T cell-mediated immune responses (86).

## Strategies to enhance immunogenicity of particulate vaccines

The ultimate aim of vaccination is to induce protective immunity against pathogens (10). The addition of adjuvants can further enhance and/or modulate the immunogenicity of particulate vaccines resulting in enhanced vaccine performance (1, 84). Induction of protective immunity requires effective priming of T and B cells (10, 59, 87). The activation and effector phases of T cell-mediated responses require two signals, “signal one” through T cell receptor recognition of peptide-MHC complexes and “signal two” *via* costimulatory receptors on T cells. Both signals are provided by APCs that encounter pathogens (1). Adjuvants can stimulate the activation and maturation of APCs, such as DCs, and thus enhance the expression of MHC and co-stimulatory molecules, which are essential for the induction of adaptive immunity (1, 10). Adjuvants are usually categorized as the delivery system (such as lipid vesicles) or immunostimulatory molecules (such as PRR ligands) based on their proposed mechanism. Most adjuvants possess both properties (88). For example, alum salts promote Th2-type immunity and B cell differentiation, leading to tremendous antibody production (78). Adjuvant System 04 (AS04) is a combination adjuvant, consisting of alum salts and TLR4 ligand monophosphoryl lipid A, which respectively upregulate potent humoral (Th2) and cellular (Th1) immune responses (**Figure 2**) (88).

As PRR ligands enable innate recognition by PRR, such as TLRs, present on DCs, the fabrication of particulate vaccines with PRR ligands can lead to targeted delivery to DCs and enhance the effectiveness of particulate vaccines (89). This enhancement is achieved by inducing “signal two” through the expression of co-stimulatory molecules on DCs, which augments T-cell responses (89). Thus coupling PRR ligands with antigens confer innate activation and antigenic stimulation to the same DC that uptakes the particulate vaccine. This lead to the generation of both signals, MHC-peptide complex and co-stimulatory signal, required for T-cell activation (1). In addition, targeted delivery to DCs can alternatively be achieved through the co-delivery of the CD40 ligand with antigen. The ligation of the CD40 ligand with the CD40 receptor on DCs serves as a key co-stimulator for DCs maturation and induction of CD4^+^ T cell responses (90).

The ability of DCs to present extracellular antigens in the context of MHC class I, a phenomenon called cross-presentation, can be an ideal target for the targeted delivery of vaccine antigens (20). Adjuvants such as MF59 and QS-21 in combination with TLR ligands can facilitate cytosolic or vacuole pathways of cross-presentation (91). In addition, cell-penetrating peptides (CPPs) also known as protein translocation domains or membrane translocating sequences, are small peptides with strong membrane permeability. CPPs are comprised of 6 to 30 amino acid residues and majority of them are basic amino acid residues, leading to an overall positive net charge (92–94). Recently the use of arginine- and lysine-rich CPPs in conjunction with particulate vaccines promotes the MHC class I pathway cross-presentation effectively against viral and tumor antigens (20, 92, 93). Moreover, DC targeting peptides (DCpep) are strongly targeted to DCs. They can improve vaccine capture efficiency, and promote DCs maturation, cytokine secretion, and T cell proliferation. Vaccines with DCpep can significantly induce stronger immune responses than the vaccines without DCpep (95). For example, Clec9A is produced on various DC subsets, such as mouse CD8a^+^ DCs and CD103^+^ DCs, and responsible for antigen cross-presentation. CBP-12 has high affinity with Clec9A on DCs. Vaccines with CBP-12 has been shown to elicit both strong cytotoxic CD8^+^ T cell and antibody responses (96, 97).

## Particulate vaccine fabrication strategies

Post-translational modifications (PTMs) regulate function of proteins including antigens and contribute to their immunological properties and stability (98). A study showed that PTMs are often required for antigens to induce functional immune responses (98). Over the last decade, an increasing number of PTMs have been detected and characterized in *E. coli*, and most PTMs are rarely found in bacterial antigens with the majority of modified antigens having a low sub-stoichiometric degree of modification (99). As a result, retaining the conformation and function of eukaryotic multi-domain antigens produced in *E. coli* is more challenging (100).

Although the *E. coli* expression system is commonly used to produce recombinant protein vaccines, mammalian or insect cells should be considered for antigens that require high levels of PTMs that are required for vaccine purposes (98, 101). These post-translationally modified complex antigens can then be incorporated into particulate platforms using a variety of methods (**Figure 2**). Non-covalent methods include the use of peptide tags like polyhistidine, protein tags such as maltose-binding protein and glutathione- *S*-transferase, DNA-directed immobilization, and the biotin–streptavidin pair (102). One of the most powerful non-covalent biological interactions known is the binding of biotin to avidin (103). Purified post-translationally modified complex antigens that have been biotin labelled are therefore highly effective for protein capture on particulate platforms for vaccine delivery, and the biotinylated antigens are subsequently recognized by avidin/streptavidin. Although chemical biotinylation frequently results in heterogeneous products with impaired function (104), enzymatic biotinylation with *E. coli* biotin ligase (BirA) attaches biotin to the 15 amino acid avidin tag (AviTag) peptide yielding a homogeneous product with a high yield (105). Streptavidin is used as a potent immunostimulant in less immunogenic antigen-based vaccines, most notably cancer vaccines, in addition to binding to the biotinylated antigen (106).

Although the interaction between streptavidin and biotin is strong, the binding can be destroyed by molecular motors (such as FtsK) in seconds (107) or shear forces in milliseconds (108), making it challenging to implement barriers or locks in cellular systems. Vaccine stability is required not just during storage but also following injection, where the lower concentration and other circumstances, such as endosomal pH and shear stress in the circulation, may mediate the dissociation of non-covalently attached antigens from particles (109). Therefore, covalent interaction between the target antigen and carriers is considered as a distinct, more robust, and long-lasting method of attaching antigens to the surface of an antigen carrier. Covalent linkage to peptide tags can be accomplished using SortaseA, SNAP-tag, split inteins, HaloTag, click reactions and Electrostatic Interaction Locks; however, SpyCatcher/SpyTag technology is most commonly used in vaccine delivery because SpyCatcher forms intermolecular isopeptide bonds selectively and spontaneously with SpyTag without the need for additional enzymes or chemical catalysis (102, 105). Furthermore, in comparison, SpyCatcher/SpyTag chemistry is reactive at the terminal and internal sites of a protein and can improve protein stability without changing its function (110). Thus, the bacterial production host can be bioengineered to produce vaccine particles displaying SpyCatcher to enable specific immobilization of SpyTag-fused target antigens with proper PTMs produced by such as mammalian or insect cell cultures (111, 112). However, several limitations typically may restrict the use of SpyCatcher/SpyTag in vaccine delivery. The final construct contains around 17 kDa molecular scar left by SpyCatcher/SpyTag coupling unlike the sortaseA with a smaller scar or split intein with no scar. Moreover, SpyCatcher/SpyTag is derived from *Streptococcus pyogenes*. The potential immunogenicity problem related to its bacterial origin may be an issue for vaccine design (113).

## Conclusion and future perspectives

Antigens delivered in particulate form show superior immunological properties when compared to corresponding antigens in soluble forms. APCs, such as DCs, can cross-present antigens taken up in particulate form to potently activate both cytotoxic CD8^+^ and CD4^+^ T cells. Particulate vaccines are versatile as they can be formulated with adjuvants and/or bioengineered for the co-delivery of antigens with PRR ligands, CPPs, and DCpep for induction of protective immunity. Immobilizing antigens to particulate carriers significantly enhances their stability such as enabling the generation of ambient-temperature stable vaccine formulations. Bacterial production hosts are unable to produce complex antigens with high levels of PTMs. This review highlighted the advances of using various technologies *via* covalent and non-covalent attachments to incorporate antigens with PTMs on particles. Although particle vaccines possess a great promise to combat infectious diseases, there are still a number of unknowns. These include a profound understanding of how particle size, charge, and structure influence the induction of immune responses. Safety concerns, such as severe anaphylaxis and myocarditis, have also been raised due to the extensive use of some new nanoparticle vaccines, such as mRNA-LNP. Understanding the underlying mechanisms of nanoparticle vaccine properties and potential toxicity could significantly advance the rational design of prophylactic and therapeutic nanoparticle vaccines.

## Author contributions

All authors contributed to the manuscript. SC and BR conceived and revised the manuscript. All authors contributed to the article and approved the submitted version.

## References

[1] Gonzalez-Miro M Chen S Gonzaga ZJ Evert B Wibowo D Rehm BH . Polyester as antigen carrier toward particulate vaccines. Biomacromolecules (2019) 20(9):3213–32. doi: 10.1021/acs.biomac.9b00509 31122016

[2] Tao P Zhu J Mahalingam M Batra H Rao VB . Bacteriophage T4 nanoparticles for vaccine delivery against infectious diseases. Adv Drug Deliv Rev (2019) 145:57–72. doi: 10.1016/j.addr.2018.06.025 29981801 PMC6759415

[3] Seyfoori A Shokrollahi Barough M Mokarram P Ahmadi M Mehrbod P Sheidary A . Emerging advances of nanotechnology in drug and vaccine delivery against viral associated respiratory infectious diseases (Varid). Int J Mol Sci (2021) 22(13):6937. doi: 10.3390/ijms22136937 34203268 PMC8269337

[4] Pielenhofer J Sohl J Windbergs M Langguth P Radsak MP . Current progress in particle-based systems for transdermal vaccine delivery. Front Immunol (2020) 11:266. doi: 10.3389/fimmu.2020.00266 32174915 PMC7055421

[5] Snapper CM . Distinct immunologic properties of soluble versus particulate antigens. Front Immunol (2018) 9:598. doi: 10.3389/fimmu.2018.00598 29619034 PMC5871672

[6] Jhaveri J Raichura Z Khan T Momin M Omri A . Chitosan nanoparticles-insight into properties, functionalization and applications in drug delivery and theranostics. Molecules (2021) 26(2):272. doi: 10.3390/molecules26020272 33430478 PMC7827344

[7] Gala RP Bajaj L Bansal A Gomes KB Joshi D Menon I . Oral vaccine delivery: The coming age of particulate vaccines to elicit mucosal immunity. Mucosal Deliv Drugs Biol Nanoparticles Springer (2020) . p:155–75. doi: 10.1007/978-3-030-35910-2_7

[8] O’Hagan DT Lodaya RN Lofano G . The continued advance of vaccine adjuvants–’We can work it out’. In: Seminars in immunology. Elsevier (2020). doi: 10.1016/j.smim.2020.101426 33257234

[9] Evert BJ Chen S McConville R Steel RW Healer J Boddey JA . Epitope-coated polymer particles elicit neutralising antibodies against plasmodium falciparum sporozoites. NPJ Vaccines (2021) 6(1):1–12. doi: 10.1038/s41541-021-00408-2 34845267 PMC8630014

[10] Wibowo D Jorritsma SH Gonzaga ZJ Evert B Chen S Rehm BH . Polymeric nanoparticle vaccines to combat emerging and pandemic threats. Biomaterials (2021) 268:120597. doi: 10.1016/j.biomaterials.2020.120597 33360074 PMC7834201

[11] Chen S Quan DH Wang XT Sandford S Kirman JR Britton WJ . Particulate mycobacterial vaccines induce protective immunity against tuberculosis in mice. Nanomaterials (2021) 11(8):2060. doi: 10.3390/nano11082060 34443891 PMC8402087

[12] Tang S Liu Z Xu W Li Q Han T Pan D . Versatile functionalization of ferritin nanoparticles by intein-mediated trans-splicing for antigen/adjuvant co-delivery. Nano Lett (2019) 19(8):5469–75. doi: 10.1021/acs.nanolett.9b01974 31251065

[13] Huang P Wang X Liang X Yang J Zhang C Kong D . Nano-, micro-, and macroscale drug delivery systems for cancer immunotherapy. Acta Biomater (2019) 85:1–26. doi: 10.1016/j.actbio.2018.12.028 30579043

[14] Burgdorf S Kurts C . Endocytosis mechanisms and the cell biology of antigen presentation. Curr Opin Immunol (2008) 20(1):89–95. doi: 10.1016/j.coi.2007.12.002 18249105

[15] Blander JM . Regulation of the cell biology of antigen cross presentation. Annu Rev Immunol (2018) 36:717. doi: 10.1146/annurev-immunol-041015-055523 29490164 PMC6430635

[16] Taefehshokr N Yin C Heit B . Rab gtpases in the differential processing of phagocytosed pathogens versus efferocytosed apoptotic cells. Histol Histopathol (2021) 36:123–35. doi: 10.14670/HH-18-252 32990320

[17] Desjardins M . Antigen cross-presentation: Proteasome location, location, location. EMBO J (2019) 38(16):e102799. doi: 10.15252/embj.2019102799 31364184 PMC6694217

[18] Fountain A Inpanathan S Alves P Verdawala MB Botelho RJ . Phagosome maturation in macrophages: Eat, digest, adapt, and repeat. Adv Biol Regul (2021) 82:100832. doi: 10.1016/j.jbior.2021.100832 34717137

[19] Cruz FM Colbert JD Rock KL . The gtp ase Rab39a promotes phagosome maturation into mhc-I antigen-presenting compartments. EMBO J (2020) 39(2):e102020. doi: 10.15252/embj.2019102020 31821587 PMC6960445

[20] Wylie B Ong F Belhoul-Fakir H Priebatsch K Bogdawa H Stirnweiss A . Targeting cross-presentation as a route to improve the efficiency of peptide-based cancer vaccines. Cancers (Basel) (2021) 13(24). doi: 10.3390/cancers13246189 PMC8699136 34944809

[21] Donahue ND Acar H Wilhelm S . Concepts of nanoparticle cellular uptake, intracellular trafficking, and kinetics in nanomedicine. Adv Drug Deliv Rev (2019) 143:68–96. doi: 10.1016/j.addr.2019.04.008 31022434

[22] Liu Z-H Deng Z-F Lu Y Fang W-H He F . A modular and self-adjuvanted multivalent vaccine platform based on porcine circovirus virus-like nanoparticles. J Nanobiotechnol (2022) 20(1):1–17. doi: 10.1186/s12951-022-01710-4 PMC9685936 36424615

[23] Roche PA Furuta K . The ins and outs of mhc class ii-mediated antigen processing and presentation. Nat Rev Immunol (2015) 15(4):203–16. doi: 10.1038/nri3818 PMC6314495 25720354

[24] Shen H Ackerman AL Cody V Giodini A Hinson ER Cresswell P . Enhanced and prolonged cross-presentation following endosomal escape of exogenous antigens encapsulated in biodegradable nanoparticles. Immunology (2006) 117(1):78–88. doi: 10.1111/j.1365-2567.2005.02268.x 16423043 PMC1782199

[25] Singh B Maharjan S Cho K-H Cui L Park I-K Choi Y-J . Chitosan-based particulate systems for the delivery of mucosal vaccines against infectious diseases. Int J Biol Macromol (2018) 110:54–64. doi: 10.1016/j.ijbiomac.2017.10.101 29054527

[26] Nooraei S Bahrulolum H Hoseini ZS Katalani C Hajizade A Easton AJ . Virus-like particles: Preparation, immunogenicity and their roles as nanovaccines and drug nanocarriers. J Nanobiotechnol (2021) 19(1):59. doi: 10.1186/s12951-021-00806-7 PMC7905985 33632278

[27] Mejía-Méndez JL Vazquez-Duhalt R Hernández LR Sánchez-Arreola E Bach H . Virus-like particles: Fundamentals and biomedical applications. Int J Mol Sci (2022) 23(15):8579. doi: 10.3390/ijms23158579 35955711 PMC9369363

[28] Tariq H Batool S Asif S Ali M Abbasi BH . Virus-like particles: Revolutionary platforms for developing vaccines against emerging infectious diseases. Front Microbiol (2021) 12:790121. doi: 10.3389/fmicb.2021.790121 35046918 PMC8761975

[29] Biela AP Naskalska A Fatehi F Twarock R Heddle JG . Programmable polymorphism of a virus-like particle. Commun Mater (2022) 3(1):7. doi: 10.1038/s43246-022-00229-3 35284827 PMC7612486

[30] Donaldson B Lateef Z Walker GF Young SL Ward VK . Virus-like particle vaccines: Immunology and formulation for clinical translation. Expert Rev Vaccines (2018) 17(9):833–49. doi: 10.1080/14760584.2018.1516552 PMC7103734 30173619

[31] Lomonossoff GP Ponndorf D . Biotechnology approaches to modern vaccine design. In: Bamford DH Zuckerman M , editors. Encyclopedia of virology, 4th ed. Oxford: Academic Press (2021). p. 662–70.

[32] Zepeda-Cervantes J Ramírez-Jarquín JO Vaca L . Interaction between virus-like particles (Vlps) and pattern recognition receptors (Prrs) from dendritic cells (Dcs): Toward better engineering of vlps. Front Immunol (2020) 11:1100. doi: 10.3389/fimmu.2020.01100 32582186 PMC7297083

[33] Chavda VP Bezbaruah R Athalye M Parikh PK Chhipa AS Patel S . Replicating viral vector-based vaccines for covid-19: Potential avenue in vaccination arena. Viruses (2022) 14(4). doi: 10.3390/v14040759 PMC9025561 35458489

[34] Travieso T Li J Mahesh S Mello JDFRE Blasi M . The use of viral vectors in vaccine development. NPJ Vaccines (2022) 7(1):75. doi: 10.1038/s41541-022-00503-y 35787629 PMC9253346

[35] Rauch S Jasny E Schmidt KE Petsch B . New vaccine technologies to combat outbreak situations. Front Immunol (2018) 9:1963. doi: 10.3389/fimmu.2018.01963 30283434 PMC6156540

[36] Zhang C Maruggi G Shan H Li J . Advances in mrna vaccines for infectious diseases. Front Immunol (2019) 10:594. doi: 10.3389/fimmu.2019.00594 30972078 PMC6446947

[37] Park JW Lagniton PNP Liu Y Xu RH . Mrna vaccines for covid-19: What, why and how. Int J Biol Sci (2021) 17(6):1446–60. doi: 10.7150/ijbs.59233 PMC8071766 33907508

[38] Rosa SS Prazeres DMF Azevedo AM Marques MPC . Mrna vaccines manufacturing: Challenges and bottlenecks. Vaccine (2021) 39(16):2190–200. doi: 10.1016/j.vaccine.2021.03.038 PMC7987532 33771389

[39] Kim J Eygeris Y Gupta M Sahay G . Self-assembled mrna vaccines. Adv Drug Delivery Rev (2021) 170:83–112. doi: 10.1016/j.addr.2020.12.014 PMC7837307 33400957

[40] Domazet-Lošo T . Mrna vaccines: Why is the biology of retroposition ignored? Genes (Basel) (2022) 13(5). doi: 10.3390/genes13050719 PMC9141755 35627104

[41] Barda N Dagan N Cohen C Hernán MA Lipsitch M Kohane IS . Effectiveness of a third dose of the Bnt162b2 mrna covid-19 vaccine for preventing severe outcomes in Israel: An observational study. Lancet (2021) 398(10316):2093–100. doi: 10.1016/s0140-6736(21)02249-2 PMC8555967 34756184

[42] Tang P Hasan MR Chemaitelly H Yassine HM Benslimane FM Al Khatib HA . Bnt162b2 and mrna-1273 covid-19 vaccine effectiveness against the sars-Cov-2 delta variant in Qatar. Nat Med (2021) 27(12):2136–43. doi: 10.1038/s41591-021-01583-4 34728831

[43] Walter EB Talaat KR Sabharwal C Gurtman A Lockhart S Paulsen GC . Evaluation of the Bnt162b2 covid-19 vaccine in children 5 to 11 years of age. N Engl J Med (2022) 386(1):35–46. doi: 10.1056/NEJMoa2116298 34752019 PMC8609605

[44] Chen M Hu X Liu S . Next-generation nonviral vectors for mrna vaccine delivery. Macromol Chem Phys (2022) 223(5):2100443. doi: 10.1002/macp.202100443

[45] Esteban I Pastor-Quiñones C Usero L Plana M García F Leal L . In the era of mrna vaccines, is there any hope for hiv functional cure? Viruses (2021) 13(3). doi: 10.3390/v13030501 PMC8003302 33803790

[46] Fan Y-N Li M Luo Y-L Chen Q Wang L Zhang H-B . Cationic lipid-assisted nanoparticles for delivery of mrna cancer vaccine. Biomater Sci (2018) 6(11):3009–18. doi: 10.1039/C8BM00908B 30264063

[47] Pippa N Gazouli M Pispas S . Recent advances and future perspectives in polymer-based nanovaccines. Vaccines (Basel) (2021) 9(6). doi: 10.3390/vaccines9060558 PMC8226647 34073648

[48] Milovanovic M Arsenijevic A Milovanovic J Kanjevac T Arsenijevic N . Chapter 14 - nanoparticles in antiviral therapy. In: Grumezescu AM , editor. Antimicrobial nanoarchitectonics. Elsevier (2017). p. 383–410. doi: 10.1016/B978-0-323-52733-0.00014-8

[49] Rehm BHA . Bioengineering towards self-assembly of particulate vaccines. Curr Opin Biotechnol (2017) 48:42–53. doi: 10.1016/j.copbio.2017.03.018 28365472

[50] Han J Zhao D Li D Wang X Jin Z Zhao K . Polymer-based nanomaterials and applications for vaccines and drugs. Polymers (Basel) (2018) 10(1). doi: 10.3390/polym10010031 PMC6415012 30966075

[51] Guo S Fu D Utupova A Sun D Zhou M Jin Z . Applications of polymer-based nanoparticles in vaccine field. Nanotechnol Rev (2019) 8(1):143–55. doi: 10.1515/ntrev-2019-0014

[52] Sabado RL Bhardwaj N . Directing dendritic cell immunotherapy towards successful cancer treatment. Immunotherapy (2010) 2(1):37–56. doi: 10.2217/imt.09.43 20473346 PMC2867472

[53] Sartorius R Trovato M Manco R D’Apice L De Berardinis P . Exploiting viral sensing mediated by toll-like receptors to design innovative vaccines. NPJ Vaccines (2021) 6(1):127. doi: 10.1038/s41541-021-00391-8 34711839 PMC8553822

[54] Keikha R Daliri K Jebali A . The use of nanobiotechnology in immunology and vaccination. Vaccines (Basel) (2021) 9(2). doi: 10.3390/vaccines9020074 PMC7910821 33494441

[55] Vanaparthy R Mohan G Vasireddy D Atluri P . Review of covid-19 viral vector-based vaccines and covid-19 variants. Infez Med (2021) 29(3):328–38. doi: 10.53854/liim-2903-3 PMC8805485 35146337

[56] Humphreys IR Sebastian S . Novel viral vectors in infectious diseases. Immunology (2018) 153(1):1–9. doi: 10.1111/imm.12829 28869761 PMC5721250

[57] Lee CS Bishop ES Zhang R Yu X Farina EM Yan S . Adenovirus-mediated gene delivery: Potential applications for gene and cell-based therapies in the new era of personalized medicine. Genes Dis (2017) 4(2):43–63. doi: 10.1016/j.gendis.2017.04.001 28944281 PMC5609467

[58] Deng S Liang H Chen P Li Y Li Z Fan S . Viral vector vaccine development and application during the covid-19 pandemic. Microorganisms (2022) 10(7):1450. doi: 10.3390/microorganisms10071450 35889169 PMC9317404

[59] Huang HY Liao HY Chen X Wang SW Cheng CW Shahed-Al-Mahmud M . Vaccination with sars-Cov-2 spike protein lacking glycan shields elicits enhanced protective responses in animal models. Sci Transl Med (2022) 14(639):eabm0899. doi: 10.1126/scitranslmed.abm0899 35230146 PMC9802656

[60] Hogan MJ Pardi N . Mrna vaccines in the covid-19 pandemic and beyond. Annu Rev Med (2022) 73:17–39. doi: 10.1146/annurev-med-042420-112725 34669432

[61] Heine A Juranek S Brossart P . Clinical and immunological effects of mrna vaccines in malignant diseases. Mol Cancer (2021) 20(1):52. doi: 10.1186/s12943-021-01339-1 33722265 PMC7957288

[62] Rijkers GT Weterings N Obregon-Henao A Lepolder M Dutt TS van Overveld FJ . Antigen presentation of mrna-based and virus-vectored sars-Cov-2 vaccines. Vaccines (Basel) (2021) 9(8). doi: 10.3390/vaccines9080848 PMC8402319 34451973

[63] Bettini E Locci M . Sars-Cov-2 mrna vaccines: Immunological mechanism and beyond. Vaccines (Basel) (2021) 9(2). doi: 10.3390/vaccines9020147 PMC7918810 33673048

[64] Fang E Liu X Li M Zhang Z Song L Zhu B . Advances in covid-19 mrna vaccine development. Signal Transduct Target Ther (2022) 7(1):94. doi: 10.1038/s41392-022-00950-y 35322018 PMC8940982

[65] Linares-Fernández S Lacroix C Exposito J-Y Verrier B . Tailoring mrna vaccine to balance Innate/Adaptive immune response. Trends Mol Med (2020) 26(3):311–23. doi: 10.1016/j.molmed.2019.10.002 31699497

[66] Nitika Wei J Hui A-M . The development of mrna vaccines for infectious diseases: Recent updates. Infect Drug Resistance (2021) 14:5271–85. doi: 10.2147/idr.s341694 PMC8668227 34916811

[67] Le T Sun C Chang J Zhang G Yin X . Mrna vaccine development for emerging animal and zoonotic diseases. Viruses (2022) 14(2):401. doi: 10.3390/v14020401 35215994 PMC8877136

[68] Chaudhary N Weissman D Whitehead KA . Mrna vaccines for infectious diseases: Principles, delivery and clinical translation. Nat Rev Drug Discovery (2021) 20(11):817–38. doi: 10.1038/s41573-021-00283-5 PMC8386155 34433919

[69] Kowalski PS Rudra A Miao L Anderson DG . Delivering the messenger: Advances in technologies for therapeutic mrna delivery. Mol Ther (2019) 27(4):710–28. doi: 10.1016/j.ymthe.2019.02.012 PMC6453548 30846391

[70] Vu MN Kelly HG Kent SJ Wheatley AK . Current and future nanoparticle vaccines for covid-19. EBioMedicine (2021) 74:103699. doi: 10.1016/j.ebiom.2021.103699 34801965 PMC8602808

[71] Lambricht L Peres C Florindo H Préat V Vandermeulen G . Chapter ten - polymer-based nanoparticles as modern vaccine delivery systems. In: Skwarczynski M Toth I , editors. Micro and nanotechnology in vaccine development. William Andrew Publishing (2017). p. 185–203. doi: 10.1016/B978-0-323-39981-4.00010-5

[72] Song R Murphy M Li C Ting K Soo C Zheng Z . Current development of biodegradable polymeric materials for biomedical applications. Drug Design Dev Ther (2018) 12:3117–45. doi: 10.2147/dddt.s165440 PMC6161720 30288019

[73] Bose RJ Kim M Chang JH Paulmurugan R Moon JJ Koh WG . Biodegradable polymers for modern vaccine development. J Ind Eng Chem (2019) 77:12–24. doi: 10.1016/j.jiec.2019.04.044 32288512 PMC7129903

[74] Miu DM Eremia MC Moscovici M . Polyhydroxyalkanoates (Phas) as biomaterials in tissue engineering: Production, isolation, characterization. Mater (Basel) (2022) 15(4). doi: 10.3390/ma15041410 PMC8875380 35207952

[75] Lee JW Parlane NA Wedlock DN Rehm BH . Bioengineering a bacterial pathogen to assemble its own particulate vaccine capable of inducing cellular immunity. Sci Rep (2017) 7:41607. doi: 10.1038/srep41607 28150705 PMC5288705

[76] Gonzaga ZJC Chen S Lehoux M Segura M Rehm BHA . Engineering antigens to assemble into polymer particle vaccines for prevention of streptococcus suis infection. Vaccines (2021) 9(12):1386. doi: 10.3390/vaccines9121386 34960132 PMC8709461

[77] Evert BJ Chen S McConville R Steel RWJ Healer J Boddey JA . Epitope-coated polymer particles elicit neutralising antibodies against plasmodium falciparum sporozoites. NPJ Vaccines (2021) 6(1):141. doi: 10.1038/s41541-021-00408-2 34845267 PMC8630014

[78] Chen S Evert B Adeniyi A Salla-Martret M Lua LH-L Ozberk V . Ambient temperature stable, scalable covid-19 polymer particle vaccines induce protective immunity. Adv Healthcare Mater (2022) 11(3):2102089. doi: 10.1002/adhm.202102089 PMC8652985 34716678

[79] Gonzaga ZJC Merakou C DiGiandomenico A Priebe GP Rehm BHA . A pseudomonas aeruginosa-derived particulate vaccine protects against p. aeruginosa infection. Vaccines (2021) 9(7):803. doi: 10.3390/vaccines9070803 34358220 PMC8309987

[80] Gonzaga ZJC Zhang J Rehm BH . Intranasal delivery of antigen-coated polymer particles protects against pseudomonas aeruginosa infection. ACS Infect Dis (2022) 8(4):744–56. doi: 10.1021/acsinfecdis.1c00434 35238554

[81] Parlane NA Grage K Lee JW Buddle BM Denis M Rehm BHA . Production of a particulate hepatitis c vaccine candidate by an engineered lactococcus lactis strain. Appl Environ Microbiol (2011) 77(24):8516–22. doi: 10.1128/AEM.06420-11 PMC3233089 21984246

[82] Parlane NA Grage K Mifune J Basaraba RJ Wedlock DN Rehm BHA . Vaccines displaying mycobacterial proteins on biopolyester beads stimulate cellular immunity and induce protection against tuberculosis. Clin Vaccine Immunol (2012) 19(1):37–44. doi: 10.1128/CVI.05505-11 22072720 PMC3255957

[83] González-Miro M Rodríguez-Noda L Fariñas-Medina M García-Rivera D Vérez-Bencomo V Rehm BHA . Self-assembled particulate psaa as vaccine against streptococcus pneumoniae infection. Heliyon (2017) 3(4):e00291. doi: 10.1016/j.heliyon.2017.e00291 28435909 PMC5390691

[84] González-Miró M Rodríguez-Noda LM Fariñas-Medina M Cedré-Marrero B Madariaga-Zarza S Zayas-Vignier C . Bioengineered polyester beads Co-displaying protein and carbohydrate-based antigens induce protective immunity against bacterial infection. Sci Rep (2018) 8(1):1888. doi: 10.1038/s41598-018-20205-7 29382864 PMC5789850

[85] Sheffee NS Rubio-Reyes P Mirabal M Calero R Carrillo-Calvet H Chen S . Engineered mycobacterium tuberculosis antigen assembly into core-shell nanobeads for diagnosis of tuberculosis. Nanomed: Nanotechnol Biol Med (2021) 34:102374. doi: 10.1016/j.nano.2021.102374 33675981

[86] Chen S Quan DH Sam G Ozberk V Wang XT Halfmann P . Assembly of immunogenic protein particles toward advanced synthetic vaccines. Small (2022). doi: 10.1002/smll.202205819 36564365

[87] Singh A . Eliciting b cell immunity against infectious diseases using nanovaccines. Nat Nanotechnol (2021) 16(1):16–24. doi: 10.1038/s41565-020-00790-3 33199883 PMC7855692

[88] Pulendran B Arunachalam P S O’Hagan DT . Emerging concepts in the science of vaccine adjuvants. Nat Rev Drug Discovery (2021) 20(6):454–75. doi: 10.1038/s41573-021-00163-y PMC8023785 33824489

[89] Kaur A Baldwin J Brar D Salunke DB Petrovsky N . Toll-like receptor (Tlr) agonists as a driving force behind next-generation vaccine adjuvants and cancer therapeutics. Curr Opin Chem Biol (2022) 70:102172. doi: 10.1016/j.cbpa.2022.102172 35785601

[90] Sharma PK Dmitriev IP Kashentseva EA Raes G Li L Kim SW . Development of an adenovirus vector vaccine platform for targeting dendritic cells. Cancer Gene Ther (2018) 25(1):27–38. doi: 10.1038/s41417-017-0002-1 29242639 PMC5972836

[91] Mosca F Tritto E Muzzi A Monaci E Bagnoli F Iavarone C . Molecular and cellular signatures of human vaccine adjuvants. Proc Natl Acad Sci (2008) 105(30):10501–6. doi: 10.1073/pnas.0804699105 PMC2483233 18650390

[92] Backlund CM Holden RL Moynihan KD Garafola D Farquhar C Mehta NK . Cell-penetrating peptides enhance peptide vaccine accumulation and persistence in lymph nodes to drive immunogenicity. Proc Natl Acad Sci (2022) 119(32):e2204078119. doi: 10.1073/pnas.2204078119 35914154 PMC9371699

[93] Silva S Almeida AJ Vale N . Combination of cell-penetrating peptides with nanoparticles for therapeutic application: A review. Biomolecules (2019) 9(1):22. doi: 10.3390/biom9010022 30634689 PMC6359287

[94] Hao M Zhang L Chen P . Membrane internalization mechanisms and design strategies of arginine-rich cell-penetrating peptides. Int J Mol Sci (2022) 23(16):9038. doi: 10.3390/ijms23169038 36012300 PMC9409441

[95] Xia T Yang H Guo Y Guo T Xin L Jiang Y . Human dendritic cell targeting peptide can be targeted to porcine dendritic cells to improve antigen capture efficiency to stimulate stronger immune response. Front Immunol (2022) 13:950597. doi: 10.3389/fimmu.2022.950597 36059519 PMC9437479

[96] Gou S Wang S Liu W Chen G Zhang D Du J . Adjuvant-free peptide vaccine targeting Clec9a on dendritic cells can induce robust antitumor immune response through Syk/Il-21 axis. Theranostics (2021) 11(15):7308. doi: 10.7150/thno.56406 34158852 PMC8210616

[97] Yan Z Wu Y Du J Li G Wang S Cao W . A novel peptide targeting Clec9a on dendritic cell for cancer immunotherapy. Oncotarget (2016) 7(26):40437. doi: 10.18632/oncotarget.9624 27250027 PMC5130018

[98] Ojha R Prajapati VK . Cognizance of posttranslational modifications in vaccines: A way to enhanced immunogenicity. J Cell Physiol (2021) 236(12):8020–34. doi: 10.1002/jcp.30483 PMC8427110 34170014

[99] Macek B Forchhammer K Hardouin J Weber-Ban E Grangeasse C Mijakovic I . Protein post-translational modifications in bacteria. Nat Rev Microbiol (2019) 17(11):651–64. doi: 10.1038/s41579-019-0243-0 31485032

[100] Rosano GL Ceccarelli EA . Recombinant protein expression in escherichia coli: Advances and challenges. Front Microbiol (2014) 5:172/BIBTEX(APR). doi: 10.3389/FMICB.2014.00172/BIBTEX 24860555 PMC4029002

[101] Nascimento IP Leite LCC . Recombinant vaccines and the development of new vaccine strategies. Braz J Med Biol Res (2012) 45(12):1102. doi: 10.1590/S0100-879X2012007500142 22948379 PMC3854212

[102] Meldal M Schoffelen S . Recent advances in covalent, site-specific protein immobilization. F1000Research (2016) 5. doi: 10.12688/F1000RESEARCH.9002.1 PMC5022707 27785356

[103] De Boer E Rodriguez P Bonte E Krijgsveldt J Katsantoni E Heckt A . Efficient biotinylation and single-step purification of tagged transcription factors in mammalian cells and transgenic mice. Proc Natl Acad Sci United States America (2003) 100(13):7480–5. doi: 10.1073/PNAS.1332608100/SUPPL_FILE/2608FIG5NEW.JPG PMC164612 12802011

[104] Predonzani A Arnoldi F López-Requena A Burrone OR . *In vivo* site-specific biotinylation of proteins within the secretory pathway using a single vector system. BMC Biotechnol (2008) 8(1):1–11. doi: 10.1186/1472-6750-8-41/FIGURES/6 18423015 PMC2373293

[105] Fairhead M Howarth M . Site-specific biotinylation of purified proteins using bira. Methods Mol Biol (Clifton NJ) (2015) 1266:171. doi: 10.1007/978-1-4939-2272-7_12 PMC4304673 25560075

[106] Jain A Cheng K . The principles and applications of avidin-based nanoparticles in drug delivery and diagnosis. J Controlled Release (2017) 245:27–40. doi: 10.1016/j.jconrel.2016.11.016 PMC5222781 27865853

[107] Chivers CE Crozat E Chu C Moy VT Sherratt DJ Howarth M . A streptavidin variant with slower biotin dissociation and increased mechanostability. Nat Methods (2010) 7(5):391–3. doi: 10.1038/nmeth.1450 PMC2862113 20383133

[108] Perumal SK Raney KD Benkovic SJ . Analysis of the DNA translocation and unwinding activities of T4 phage helicases. Methods (2010) 51(3):277–88. doi: 10.1016/j.ymeth.2010.02.011 PMC2919206 20170733

[109] Brune KD Howarth M . New routes and opportunities for modular construction of particulate vaccines: Stick, click, and glue. Front Immunol (2018) 9:1432. doi: 10.3389/fimmu.2018.01432 29997617 PMC6028521

[110] Zakeri B Fierer JO Celik E Chittock EC Schwarz-Linek U Moy VT . Peptide tag forming a rapid covalent bond to a protein, through engineering a bacterial adhesin. Proc Natl Acad Sci (2012) 109(12):E690–E7. doi: 10.1073/pnas.1115485109 PMC3311370 22366317

[111] Lin Z Lin Q Li J Pistolozzi M Zhao L Yang X . Spy chemistry-enabled protein directional immobilization and protein purification. Biotechnol Bioengineering (2020) 117(10):2923–32. doi: 10.1002/BIT.27460 32543719

[112] Tian J Jia R Wenge D Sun H Wang Y Chang Y . One-step purification and immobilization of recombinant proteins using Spytag/Spycatcher chemistry. Biotechnol Lett (2021) 43(5):1075–87. doi: 10.1007/S10529-021-03098-X 33591462

[113] Sutherland AR Alam MK Geyer CR . Post-translational assembly of protein parts into complex devices by using Spytag/Spycatcher protein ligase. ChemBioChem (2019) 20(3):319–28. doi: 10.1002/cbic.201800538 30358052

